# Supplementary material for: Feasibility and reliability of online vs in-person cognitive testing in healthy older people
Source: PLoS One. 2024 Aug 20;19(8):e0309006. doi: 10.1371/journal.pone.0309006 (PMC11335153; doi:10.1371/journal.pone.0309006)
Supplement: S1 File — S1 Table. Cognitive battery tasks. S2A Table: Full model of MRA between Reaction Time and demographic characteristics. S2B Table: Full model of MRA between TMT-A performance and demographic characteristics. S2C Table: Full model of MRA between TMT-B performance and demographic characteristics. S2D Table: Full model of MRA between Spatial Working Memory performance and demographic characteristics. S2E Table: Full model of MRA between Episodic Memory performance and demographic characteristics. S2F Table: Full model of MRA between Go/No-Go performance and demographic characteristics. S2G Table: Full model of MRA between Allocentric Orientation performance and demographic characteristics. S2H Table: Full model of MRA between Egocentric Orientation and demographic characteristics. S2I Table: Full model of MRA between global cognitive performance and demographic characteristics. S3 Figs: Residuals distribution for significant multiple regression results. S4 Table: Cognitive task performance compared across devices used for testing. S5 Table: Navigation variables correlation with the Driving, Orientation, and Navigation score. (ZIP) [file pone.0309006.s001.zip › S5 Table. Navigation variables correlation with the Driving, Orientation, and Navigation score.docx]

**S5 Appendix:**

**S5 Table: Navigation variables correlation with the Driving, Orientation, and Navigation score**

| **Variable** | **Pearson’s r** |
| --- | --- |
| Santa Barbara Sense of Direction | **0.67***** |
| Allocentric Navigation | -0.24 |
| Egocentric Navigation | 0.01 |

^a^*p < .05, **p < .01, ***p < .001

Pearson’s correlations were conducted to establish the association between subjective navigation performance using the novel Driving, Orientation, and Navigation (DON) questionnaire with the established Santa Barbara Sense of Direction Scale (SBSOD) and objective spatial orientation measures – allocentric and egocentric orientation. There was a significant positive association between DON and SBSOD ratings to a moderate to strong correlation, r(30) = 0.67, *p* < .001.
