# Supplementary material for: Feasibility and reliability of online vs in-person cognitive testing in healthy older people
Source: PLoS One. 2024 Aug 20;19(8):e0309006. doi: 10.1371/journal.pone.0309006 (PMC11335153; doi:10.1371/journal.pone.0309006)
Supplement: S1 File — S1 Table. Cognitive battery tasks. S2A Table: Full model of MRA between Reaction Time and demographic characteristics. S2B Table: Full model of MRA between TMT-A performance and demographic characteristics. S2C Table: Full model of MRA between TMT-B performance and demographic characteristics. S2D Table: Full model of MRA between Spatial Working Memory performance and demographic characteristics. S2E Table: Full model of MRA between Episodic Memory performance and demographic characteristics. S2F Table: Full model of MRA between Go/No-Go performance and demographic characteristics. S2G Table: Full model of MRA between Allocentric Orientation performance and demographic characteristics. S2H Table: Full model of MRA between Egocentric Orientation and demographic characteristics. S2I Table: Full model of MRA between global cognitive performance and demographic characteristics. S3 Figs: Residuals distribution for significant multiple regression results. S4 Table: Cognitive task performance compared across devices used for testing. S5 Table: Navigation variables correlation with the Driving, Orientation, and Navigation score. (ZIP) [file pone.0309006.s001.zip › S2D Table. Full model of MRA between Spatial Working Memory performance and demographic characteristics.docx]

**S2D Table: Full model of MRA between Spatial Working Memory performance and demographic characteristics**

| **Effect** | ***B*** | ***SE*** | **95% CI** | | ***p*** |
| --- | --- | --- | --- | --- | --- |
|  |  |  | **LL** | **UL** |  |
| (Intercept) | 5.31 | 2.66 | -0.15 | 10.77 | 0.06 |
| Traditional test score | 0.23 | 0.18 | -0.15 | 0.61 | 0.22 |
| Age | -0.07 | 0.04 | -0.15 | 0.01 | 0.07 |
| Sex | -0.25 | 0.35 | -0.98 | 0.47 | 0.48 |
| Education | -0.02 | 0.05 | -0.11 | 0.08 | 0.70 |

^a^Standardised beta coefficients displayed.
^b^ Traditional test score = Corsi block tapping test.
