# Supplementary material for: Feasibility and reliability of online vs in-person cognitive testing in healthy older people
Source: PLoS One. 2024 Aug 20;19(8):e0309006. doi: 10.1371/journal.pone.0309006 (PMC11335153; doi:10.1371/journal.pone.0309006)
Supplement: S1 File — S1 Table. Cognitive battery tasks. S2A Table: Full model of MRA between Reaction Time and demographic characteristics. S2B Table: Full model of MRA between TMT-A performance and demographic characteristics. S2C Table: Full model of MRA between TMT-B performance and demographic characteristics. S2D Table: Full model of MRA between Spatial Working Memory performance and demographic characteristics. S2E Table: Full model of MRA between Episodic Memory performance and demographic characteristics. S2F Table: Full model of MRA between Go/No-Go performance and demographic characteristics. S2G Table: Full model of MRA between Allocentric Orientation performance and demographic characteristics. S2H Table: Full model of MRA between Egocentric Orientation and demographic characteristics. S2I Table: Full model of MRA between global cognitive performance and demographic characteristics. S3 Figs: Residuals distribution for significant multiple regression results. S4 Table: Cognitive task performance compared across devices used for testing. S5 Table: Navigation variables correlation with the Driving, Orientation, and Navigation score. (ZIP) [file pone.0309006.s001.zip › S4 Table. Cognitive task performance compared across devices used for testing.docx]

**S4 Appendix:**

**S4 Table:** **Cognitive task performance compared across devices used for testing**

| **Variable** | **PC** | **Laptop** | **Tablet** | ***F*** |
| --- | --- | --- | --- | --- |
| Reaction Time (ms) | 316.37 (34.57) | 345.79 (80.33) | 415.03 (63.13) | *4.410** |
| Trail-Making Test A (s) | 31.28 (9.52) | 34.88 (13.63) | 35.54 (8.70) | 1.013 |
| Trail-Making Test B (s) | 50.27 (19.88) | 48.77 (20.82) | 53.59 (16.38) | 0.184 |
| Spatial Working Memory | 5.31 (0.85) | 5.38 (1.04) | 5.83 (1.17) | 0.691 |
| Episodic Memory | 89.38 (10.00) | 90.17 (6.21) | 94.66 (7.78) | 0.919 |
| Go/No-Go | 1.46 (1.76) | 1.54 (1.71) | 0.00 (0.00) | 2.203 |
| Allocentric Orientation | 2.77 (1.70) | 3.04 (1.29) | 4.47 (1.64) | 2.277 |
| Egocentric Orientation | 60.12 (34.50) | 45.67 (33.11) | 40.97 (6.38) | 0.737 |
| Global cognition | 0.12 (0.45) | 0.16 (0.45) | 0.08 (0.37) | 0.051 |

^a^ Covariates for ANCOVAs: RT (Age), TMT-A (Age), TMT-B (Age), SWM (Age), EM (Age + Sex), GNG, AO (Age + Sex), EO (Age + Sex), Global cognition (Age).

There was a statistically significant difference with Reaction Time across devices used, *F*(2, 27) = 4.410, *p*  = 0.02, η_p_^2^ = .25. Tukey’s post-hoc pairwise comparisons revealed that individuals using PCs (*M* = 316.37, SD = 34.57) demonstrated a faster reaction time individuals using tablets (*M* = 415.03, SD = 63.13).
