# Supplementary figures and images for: Feasibility and reliability of online vs in-person cognitive testing in healthy older people
Source: PLoS One. 2024 Aug 20;19(8):e0309006. doi: 10.1371/journal.pone.0309006 (PMC11335153; doi:10.1371/journal.pone.0309006)

**S3 Figures: Residuals distribution for significant multiple regression results**

**
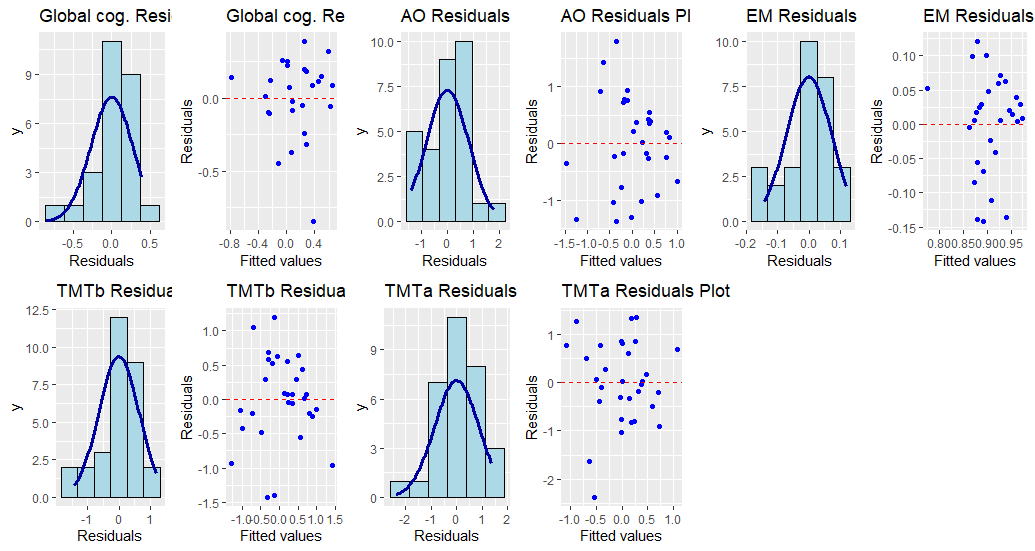
**

Supplement: S1 File — S1 Table. Cognitive battery tasks. S2A Table: Full model of MRA between Reaction Time and demographic characteristics. S2B Table: Full model of MRA between TMT-A performance and demographic characteristics. S2C Table: Full model of MRA between TMT-B performance and demographic characteristics. S2D Table: Full model of MRA between Spatial Working Memory performance and demographic characteristics. S2E Table: Full model of MRA between Episodic Memory performance and demographic characteristics. S2F Table: Full model of MRA between Go/No-Go performance and demographic characteristics. S2G Table: Full model of MRA between Allocentric Orientation performance and demographic characteristics. S2H Table: Full model of MRA between Egocentric Orientation and demographic characteristics. S2I Table: Full model of MRA between global cognitive performance and demographic characteristics. S3 Figs: Residuals distribution for significant multiple regression results. S4 Table: Cognitive task performance compared across devices used for testing. S5 Table: Navigation variables correlation with the Driving, Orientation, and Navigation score. (ZIP) [file pone.0309006.s001.zip › S3 Figures. Residuals distribution for significant multiple regression results.docx]
