# Supplementary material for: Feasibility and reliability of online vs in-person cognitive testing in healthy older people
Source: PLoS One. 2024 Aug 20;19(8):e0309006. doi: 10.1371/journal.pone.0309006 (PMC11335153; doi:10.1371/journal.pone.0309006)
Supplement: S1 Data — (DOCX) [file pone.0309006.s002.docx]

Supporting Information

**S1 Appendix**

**S1 Table.** **Cognitive battery tasks**

| **Task:** | **Domain:** | **Description:** |
| --- | --- | --- |
| Reaction time | Visuomotor speed | Participants respond (via keyboard, touchscreen) as quickly as possible to a repeating stimulus that appears on the screen. |
| Trail-Making Test -A | Processing speed | Participants connect a set of 25 numerically arranged points in ascending order as quickly as possible. |
| Trail-Making Test -B | Executive functioning | Participants connect a set of 25 points in ascending order alternating between numbers and letters. |
| Spatial Span – Backwards | Spatial working memory | Based on the Corsi block test, participants are presented with an array of geometric shapes that light up in a different sequential order per trial. After each trial, the participant relays the previous sequence in reverse order. The difficulty increases systematically from two box to nine box sequences. The task aborts if participants incorrectly relay two sequences in the same trial sequence length. |
| Recognition & Source Memory | Episodic Memory | Participants initially view a set of everyday objects that appear consecutively at in different places (top, bottom, left, right) of the screen in a learning phase. After a break, participants are tested on whether they correctly recognise pictures they previously learnt in a recognition memory test and are then asked to locate the position they appeared on the screen in a source memory test. 30 pictures are presented in the test session. |
| Go/No-Go | Attentional control | Participants are asked to press a key when a circle displays (Go stimuli) and to inhibit responses when a triangle is displayed (No-go stimuli). The task consists of 150 stimuli presentations. |
| Fragmented Letters | Visuospatial impairment | Participants identify a single letter from the alphabet that is fragmented through a visual mask. Participants must then select the presented letter out of multiple choices. There are 10 trials in total. |
| Virtual Supermarket Task | Allocentric & Egocentric orientation | Participants view 14 randomly ordered 20-40 second clips of a trolley moving through a virtual supermarket. Each video is presented in first-person perspective and contain optic flow cues via the changing scenery as the shopping trolley moves throughout the supermarket. Following the video clip, participants are asked to indicate a direction to the starting point of the video - assessing egocentric orientation - and then are asked to draw the path presented in the video from a birds-eye view of the supermarket – assessing allocentric orientation. This task has been previously described in detail [1]. |

**S2 Appendix:**

**S2A Table: Full model of MRA between Reaction Time and demographic characteristics**

| **Effect** | ***B*** | ***SE*** | **95% CI** | | ***p*** |
| --- | --- | --- | --- | --- | --- |
|  |  |  | **LL** | **UL** |  |
| (Intercept) | 0.93 | 2.72 | -104.26 | 665.27 | 0.74 |
| Age | -0.01 | 0.04 | -0.09 | 0.06 | 0.76 |
| Sex | -0.65 | 0.36 | -1.40 | 0.09 | 0.08 |
| Education | 0.01 | 0.04 | -0.08 | 0.11 | 0.79 |

^a^Standardised beta coefficients displayed.

**S2B Table: Full model of MRA between TMT-A performance and demographic characteristics**

| **Effect** | ***B*** | ***SE*** | **95% CI** | | ***p*** |
| --- | --- | --- | --- | --- | --- |
|  |  |  | **LL** | **UL** |  |
| (Intercept) | 8.05 | 3.89 | 0.06 | 16.04 | 0.049 |
| Traditional test score | 0.20 | 0.17 | -0.16 | 0.55 | 0.27 |
| Age | -0.12 | 0.05 | -0.23 | -0.01 | 0.03 |
| Sex | 0.19 | 0.34 | -0.50 | 0.88 | 0.58 |
| Education | 0.02 | 0.04 | -0.07 | 0.11 | 0.60 |

^a^Standardised beta coefficients displayed.
^b^ Traditional test score = Paper-based TMT-A

**S2C Table: Full model of MRA between TMT-B performance and demographic characteristics**

| **Effect** | ***B*** | ***SE*** | **95% CI** | | ***p*** |
| --- | --- | --- | --- | --- | --- |
|  |  |  | **LL** | **UL** |  |
| (Intercept) | 1.87 | 3.25 | -4.81 | 8.56 | 0.57 |
| Traditional test score | 0.49 | 0.14 | 0.20 | 0.78 | 0.002 |
| Age | -0.04 | 0.04 | -0.13 | 0.05 | 0.35 |
| Sex | -0.11 | 0.26 | -0.65 | 0.42 | 0.67 |
| Education | 0.07 | 0.04 | -0.00 | 0.15 | 0.06 |

^a^Standardised beta coefficients displayed.
^b^ Traditional test score = Paper-based TMT-B

**S2D Table: Full model of MRA between Spatial Working Memory performance and demographic characteristics**

| **Effect** | ***B*** | ***SE*** | **95% CI** | | ***p*** |
| --- | --- | --- | --- | --- | --- |
|  |  |  | **LL** | **UL** |  |
| (Intercept) | 5.31 | 2.66 | -0.15 | 10.77 | 0.06 |
| Traditional test score | 0.23 | 0.18 | -0.15 | 0.61 | 0.22 |
| Age | -0.07 | 0.04 | -0.15 | 0.01 | 0.07 |
| Sex | -0.25 | 0.35 | -0.98 | 0.47 | 0.48 |
| Education | -0.02 | 0.05 | -0.11 | 0.08 | 0.70 |

^a^Standardised beta coefficients displayed.
^b^ Traditional test score = Corsi block tapping test.

**S2E Table: Full model of MRA between Episodic Memory performance and demographic characteristics**

| **Effect** | ***B*** | ***SE*** | **95% CI** | | ***p*** |
| --- | --- | --- | --- | --- | --- |
|  |  |  | **LL** | **UL** |  |
| (Intercept) | 0.71 | 0.23 | 0.25 | 1.18 | 0.004 |
| Traditional test score | 0.02 | 0.02 | -0.01 | 0.05 | 0.19 |
| Age | 0.00 | 0.00 | -0.00 | 0.01 | 0.58 |
| Sex | 0.07 | 0.03 | 0.01 | 0.13 | 0.03 |
| Education | 0.00 | 0.00 | -0.01 | 0.01 | 0.64 |

^a^Standardised beta coefficients displayed.
^b^ Traditional test score = ROCF-delayed recall test.

**S2F Table: Full model of MRA between Go/No-Go performance and demographic characteristics**

| **Effect** | ***B*** | ***SE*** | **95% CI** | | ***p*** |
| --- | --- | --- | --- | --- | --- |
|  |  |  | **LL** | **UL** |  |
| (Intercept) | -3.14 | 2.70 | -8.67 | 2.38 | 0.25 |
| Age | 0.03 | 0.04 | -0.05 | 0.10 | 0.45 |
| Sex | 0.58 | 0.35 | -0.15 | 1.30 | 0.12 |
| Education | 0.06 | 0.05 | -0.04 | 0.15 | 0.23 |

^a^Standardised beta coefficients displayed.

**S2G Table: Full model of MRA between Allocentric Orientation performance and demographic characteristics**

| **Effect** | ***B*** | ***SE*** | **95% CI** | | ***p*** |
| --- | --- | --- | --- | --- | --- |
|  |  |  | **LL** | **UL** |  |
| (Intercept) | 5.44 | 2.38 | 0.55 | 10.33 | 0.03 |
| Age | -0.10 | 0.03 | -0.16 | -0.03 | 0.007 |
| Sex | -0.26 | 0.32 | -0.92 | 0.41 | 0.44 |
| Education | 0.09 | 0.04 | 0.00 | 0.17 | 0.045 |

^a^Standardised beta coefficients displayed.

**S2H Table: Full model of MRA between Egocentric Orientation and demographic characteristics**

| **Effect** | ***B*** | ***SE*** | **95% CI** | | ***p*** |
| --- | --- | --- | --- | --- | --- |
|  |  |  | **LL** | **UL** |  |
| (Intercept) | -0.30 | 2.76 | -5.97 | 5.38 | 0.91 |
| Age | -0.01 | 0.03 | -0.09 | 0.07 | 0.74 |
| Sex | 0.38 | 0.37 | -0.39 | 1.15 | 0.32 |
| Education | 0.06 | 0.05 | -0.04 | 0.16 | 0.20 |

^a^Standardised beta coefficients displayed.

**S2I Table: Full model of MRA between global cognitive performance and demographic characteristics**

| **Effect** | ***B*** | ***SE*** | **95% CI** | | ***p*** |
| --- | --- | --- | --- | --- | --- |
|  |  |  | **LL** | **UL** |  |
| (Intercept) | 0.40 | 1.56 | -2.84 | 3.63 | 0.81 |
| Traditional test score | 0.11 | 0.03 | 0.04 | 0.17 | 0.003 |
| Age | -0.05 | 0.02 | -0.09 | -0.02 | 0.007 |
| Sex | -0.05 | 0.12 | -0.30 | 0.19 | 0.66 |
| Education | 0.03 | 0.02 | -0.00 | 0.06 | 0.09 |

^a^Standardised beta coefficients displayed.
^b^ Traditional test score = MoCA

**S3 Appendix:**

**S3 Figures: Residuals distribution for significant multiple regression results**

**
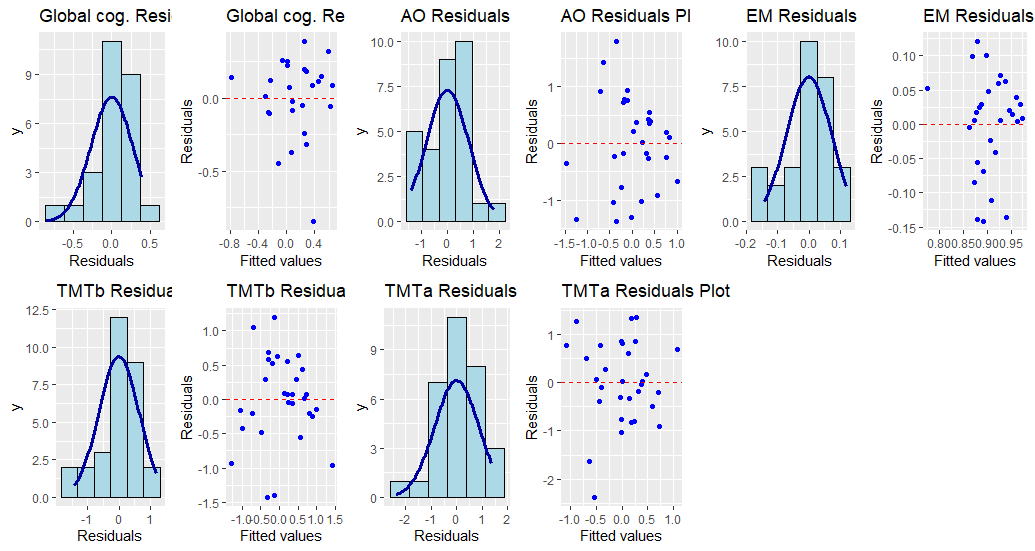
**

**S4 Appendix:**

**S4 Table:** **Cognitive task performance compared across devices used for testing**

| **Variable** | **PC** | **Laptop** | **Tablet** | ***F*** |
| --- | --- | --- | --- | --- |
| Reaction Time (ms) | 316.37 (34.57) | 345.79 (80.33) | 415.03 (63.13) | *4.410** |
| Trail-Making Test A (s) | 31.28 (9.52) | 34.88 (13.63) | 35.54 (8.70) | 1.013 |
| Trail-Making Test B (s) | 50.27 (19.88) | 48.77 (20.82) | 53.59 (16.38) | 0.184 |
| Spatial Working Memory | 5.31 (0.85) | 5.38 (1.04) | 5.83 (1.17) | 0.691 |
| Episodic Memory | 89.38 (10.00) | 90.17 (6.21) | 94.66 (7.78) | 0.919 |
| Go/No-Go | 1.46 (1.76) | 1.54 (1.71) | 0.00 (0.00) | 2.203 |
| Allocentric Orientation | 2.77 (1.70) | 3.04 (1.29) | 4.47 (1.64) | 2.277 |
| Egocentric Orientation | 60.12 (34.50) | 45.67 (33.11) | 40.97 (6.38) | 0.737 |
| Global cognition | 0.12 (0.45) | 0.16 (0.45) | 0.08 (0.37) | 0.051 |

^a^ Covariates for ANCOVAs: RT (Age), TMT-A (Age), TMT-B (Age), SWM (Age), EM (Age + Sex), GNG, AO (Age + Sex), EO (Age + Sex), Global cognition (Age).

There was a statistically significant difference with Reaction Time across devices used, *F*(2, 27) = 4.410, *p*  = 0.02, η_p_^2^ = .25. Tukey’s post-hoc pairwise comparisons revealed that individuals using PCs (*M* = 316.37, SD = 34.57) demonstrated a faster reaction time individuals using tablets (*M* = 415.03, SD = 63.13).

**S5 Appendix:**

**S5 Table: Navigation variables correlation with the Driving, Orientation, and Navigation score**

| **Variable** | **Pearson’s r** |
| --- | --- |
| Santa Barbara Sense of Direction | **0.67***** |
| Allocentric Navigation | -0.24 |
| Egocentric Navigation | 0.01 |

^a^*p < .05, **p < .01, ***p < .001

Pearson’s correlations were conducted to establish the association between subjective navigation performance using the novel Driving, Orientation, and Navigation (DON) questionnaire with the established Santa Barbara Sense of Direction Scale (SBSOD) and objective spatial orientation measures – allocentric and egocentric orientation. There was a significant positive association between DON and SBSOD ratings to a moderate to strong correlation, r(30) = 0.67, *p* < .001.

**References:**

1. Tu S, Wong S, Hodges JR, Irish M, Piguet O, Hornberger M. Lost in spatial translation - A novel tool to objectively assess spatial disorientation in Alzheimer’s disease and frontotemporal dementia. Cortex [Internet]. 2015 Jun 1 [cited 2024 Jan 11];67:83–94. Available from: https://pubmed.ncbi.nlm.nih.gov/25913063/
